# Embryonic stem cells in scaffold-free three-dimensional cell culture: osteogenic differentiation and bone generation

**DOI:** 10.1186/1746-160X-7-12

**Published:** 2011-07-14

**Authors:** Jörg Handschel, Christian Naujoks, Rita Depprich, Lydia Lammers, Norbert Kübler, Ulrich Meyer, Hans-Peter Wiesmann

**Affiliations:** 1 Department for Cranio- and Maxillofacial Surgery, Heinrich-Heine-Universität, Moorenstr. 5, D- 40225 Düsseldorf, Germany; 2 Department for Cranio- and Maxillofacial Surgery, Westfälische-Wilhelms-Universität, Waldeyerstr. 30, D-48149 Münster, Germany; 3 Department for Material Science, Technical University of Dresden, Helmholtzstr. 7, D-01062 Dresden, Germany

**Keywords:** Embryonal stem cell, osteogenic tissue engineering, three-dimensional culture technique, scaffold free tissue, hydroxyl apatite

## Abstract

Extracorporeal formation of mineralized bone-like tissue is still an unsolved challenge in tissue engineering. Embryonic stem cells may open up new therapeutic options for the future and should be an interesting model for the analysis of fetal organogenesis. Here we describe a technique for culturing embryonic stem cells (ESCs) in the absence of artificial scaffolds which generated mineralized miromasses. Embryonic stem cells were harvested and osteogenic differentiation was stimulated by the addition of dexamethasone, ascorbic acid, and ß-glycerolphosphate (DAG). After three days of cultivation microspheres were formed. These spherical three-dimensional cell units showed a peripheral zone consisting of densely packed cell layers surrounded by minerals that were embedded in the extracellular matrix. Alizarine red staining confirmed evidence of mineralization after 10 days of DAG stimulation in the stimulated but not in the control group. Transmission electron microscopy demonstrated scorching crystallites and collagenous fibrils as early indication of bone formation. These extracellular structures resembled hydroxyl apatite-like crystals as demonstrated by distinct diffraction patterns using electron diffraction analysis. The micromass culture technique is an appropriate model to form three-dimensional bone-like micro-units without the need for an underlying scaffold. Further studies will have to show whether the technique is applicable also to pluripotent stem cells of different origin.

## Introduction

Bony defects have various causes and often turn out to be a major therapeutic challenge. Until today, the reconstruction of bone using autologous grafts has been recognized as the gold standard because it provides biological active cells with osteoinductive properties and avoids any immunological reactions [1]. Unfortunately, the harvesting of these grafts causes donor-side defects and shows a quantitative limitation [2-4]. Artificial materials and extracorporeal tissue formation are alternative approaches for the reconstruction of bone defects, because they neither cause donor-site lesions nor is their availabilty restricted.

Bone is a highly specialized tissue of the organism which is generated by mineralization of the extracellular matrix called osteoid. Osteoblasts and osteoclasts contribute to the formation and remodelling of bone tissue. However, there are further cell types e.g. endothelia cells, which are also essential for bone formation [5]. The complex cell-driven process of bone formation starts early in the embryo and results in bone tissue with unique features that combines stiffness and elasticity with the ability to regenerate itself [6]. A key feature of bone tissue is the presence of biological active apatite crystals. These crystals were formatted by the mineralization of the extracellular matrix (osteoid) with calcium and phosphate ions. The process of mineralization can be monitored histologically by special stainings like alizarin red or ultrastructurally by transmission (TEM) and scanning electron microscopy (SEM).

Common approaches for engineering bone ex vivo are usually based on a combination of cells and scaffolds [7-9]. Even the ex vivo de novo bone building starts with the secretion of collagen via matrix vesicles followed by the mineralisation of the extracellular matrix molecules [7]. It has been reported that cells in three-dimensional cultures exert higher proliferation rates than cells cultured in monolayers, suggesting that their differentiation resembles more closely that seen in situ [10-12]. Furthermore, it is assumed that cells are more flexible to change their shape and behaviour upon specific cell signals when they are cultured in three-dimensional as compared to two-dimensional cultures [13,14].

Whereas a multitude of extracorporeal bone tissue engineering approaches have been undertaken to fabricate bone tissue ex vivo, up to now cell culture-based methods for synthesizing bone-like tissue on a structural level are still limited due to technical restrictions [15]. Here we describe that mineralized bone-like matrix is produced by osteoinduced totipotent embryonic stem cells cultured in three-dimensional micromass technique in the absence of any scaffold. The osteogenic differentiation of the cells was induced by the addition of dexamethasone, ascorbic acid, and ß-glycerolphosphate (DAG) to the medium [16,17]. The features of ossification mimic in-vivo bone formation, thus enabling matured mineralized bone matrix to be generated.

## Materials and methods

### Cell culture

A cell culture method for producing mineralized biomaterial-free, three-dimensional cell units up to 0.4 mm in diameter was established. Feeder-independent murine embryonic stem cells (ESCs) were kindly provided by K. Pfeffer (Institute for Microbiology, Heinrich Heine University of Düsseldorf, Germany). The cells were derived from the inner cell mass of blastocysts extracted from C57BL/6 mice and tested positive for the stem cell markers Pouf1 (alias Oct4) and Foxd3 [18]. Cells were cultured in Dulbecco's modified Eagle medium (DMEM, Gibco) supplemented with penicillin (100 U/ml, Grünenthal), streptomycin (100 U/ml, Hefa-pharma), 2-mercaptoethanol (500 mM, Gibco), ultraglutamine (2 mM; Cambrex), leukemia inhibitor factor (1000 U/ml; Chemicon) and 15% fetal calf serum. The cells were split every second day and the medium was changed every day by detaching the cells with 0.25% trypsin (Pan Biotech). ESCs were detached from the plate, centrifuged and resuspended in normal growth medium (1 × 106 cells/ml).

To prevent adherence of the cells leading to the formation of monolayers, the microsphere assembly bioreactor was prepared by filling 60 μl of a solution consisting of 2% agarose in DMEM (without any supplements) into 96-well plates. After curing of the agarose solution to each well, 180 μl of cell suspension was added and the cells were incubated overnight. The old medium was replaced by equal volumes (160 μl) of control medium and control medium containing 100 nM dexamethasone, 50 μM ascorbic acid, and 10 mM β-glyerolphosphate (all from Sigma), respectivey. Thus, half of the culture chambers were incubated in the presence of dexamethasone, ascorbic acid, and DAG, (DAG (+)) to induce the osteogenic differentiation, while the other half used as a control was cultivated in medium without these stimuli (DAG (-)). Both cell populations were kept in culture for three weeks in an incubator under a humified atmosphere (37°C, 90% humidity, 5% CO_2_). The medium was changed every day. After 3, 7, 10, and 21 days one quarter of the cultivated wells with microspheres of the + and - DAG group was harvested and transferred into Petri dishes for a washing step with phosphate-buffered saline (PBS). Subsequently the preparation of the spheres for the different analysis was performed.

### Histological analysis

For histologiacal analysis, micromasses were fixed in formalin (4%) until further procession. Formaline-fixed microspheres were dehydrated in increasing ethanol concentrations (50%, 75%, 90% and 100%) and embedded in paraffin (Paraplast plus). Sections (4 μm) were mounted on Superfrost slides, deparaffinized with xylol and rehydrated in decreasing ethanol concentrations. Samples were stained with alizarine red solution (2%) to detect calcium and counterstained with toluidine blue, as mentioned in the literature. Briefly, after staining with toludine blue the slides were counterstained with alizarin red (mixture of 0.5 g alizarin red and 0.5 ml 0.28% NH3 with 45 ml distilled water (pH: 6.4)). Before the slides were finally covered with entellan, they were incubated in xylene. A descriptive analysis was performed.

### Scanning electron microscopy

For scanning electron microscopy, micromasses were fixed in glutaraldehyde (4%) followed by a washing step with 0.1 M PBS. Microspheres were dehydrated in increasing isopropanol concentrations (30%, 50%, 70%, 90%, 96%, and 100%; 30 minutes for each concentration). The critical pont drying was performed following the instructors protocol. In this procedure isopropanol was substituted for CO2 by five washing steps. After drying, the specimens were directly put on a carbon pad of a SEM-holder (Cambridge). For morphological studies, probes were sputtered with platinum, whereas for EDX analysis, samples were coated with carbon using standard techniques. Scanning electron microscopy was performed with a DSM 960 (Zeiss) microscope using an acceleration voltage of 5-15 kV.

### Transmission electron microscopy

For TEM, specimens were fixated in glutaraldehyde (2.5%) and embedded in araldite. For morphological analysis, a fixation with osmium tetroxide and glutaraldehyde was carried out. Specimens were washed three times with 0.1 M PBS for 10 minutes each. Microspheres were dehydrated in increasing isopropanol concentrations (50%, 70%, 90%, 96% and 100%; 30 minutes for each concentration) and followed by a transfer into propylene oxid. Afterwards the spheres were transferred to pure araldite by using intermediate ratios of mixtures (100% propylene oxide, 2/1 propylene oxide/araldite, 1/1, 1/2, 100% araldite). To harden the araldite the specimen were kept at 42°C for 24 hours and afterwards were sectioned with a microtome (Ultracut S, Reichert). For morphological studies ultrathin sections were stained with osmium tetroxide (OsO_4_). For ultrastructural assessment of the mineral substance no staining was performed and the water contact during preparation, particulary during sectioning, was reduced to a minimum in order to avoid dissolution or redistribution. The ultrathin slides were applied to copper grids and contrasted with uranyl acetate. Analyses were performed with an acceleration voltage of 80 kV with EM902 (Zeiss). Electron spectroscopic diffraction analysis was performed with the specimens used for the TEM. Contact time of the slides with water on the microtome was limited to a few seconds to avoid redistribution of the crystallites. Analyses were performed with an EM902 (Zeiss) microscopy using 80 kV acceleration voltage and a camera length of 650 mm. D-values for the 002 diffraction patterns were calculated according to Arnold et al. and Plate et al. [19,20].

## Results

**Figure 1:**
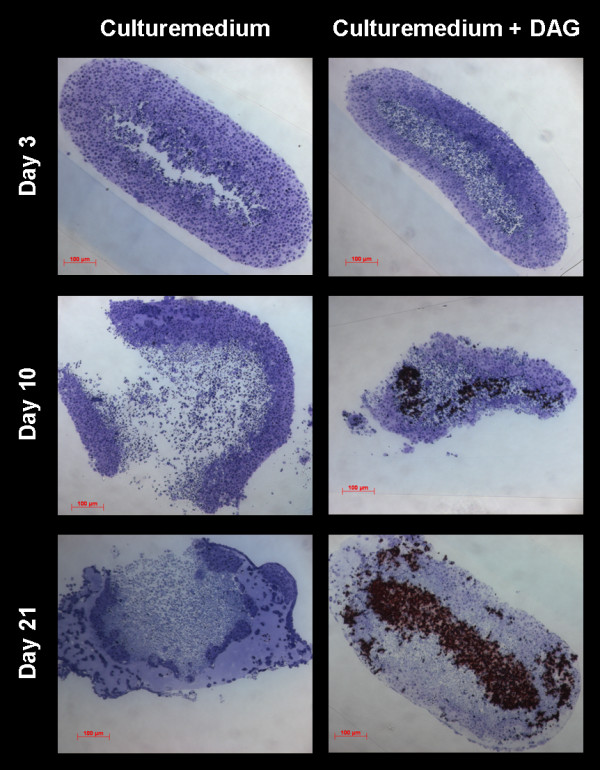
**Micromasses consisting of embryonic stem cells were cultured with or without DAG and stained with toluidine blue followed by counterstaining with alizarin red**. Shown is evidence for the mineralization in the centre of the micromasses, which were stained in red.

**Figure 2:**
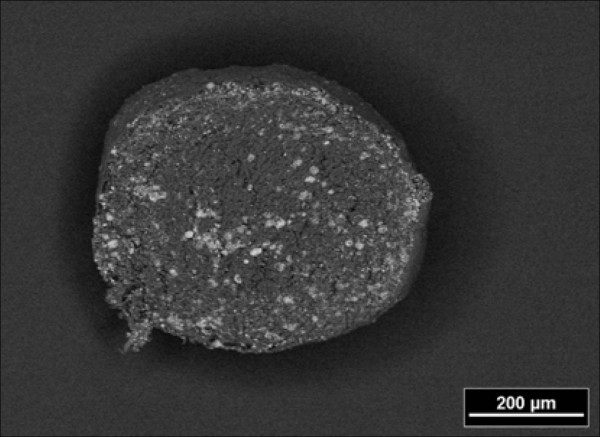
**SEM image of the mineralization in the centre of a +DAG spheres after 21 days**.

**Figure 3:**
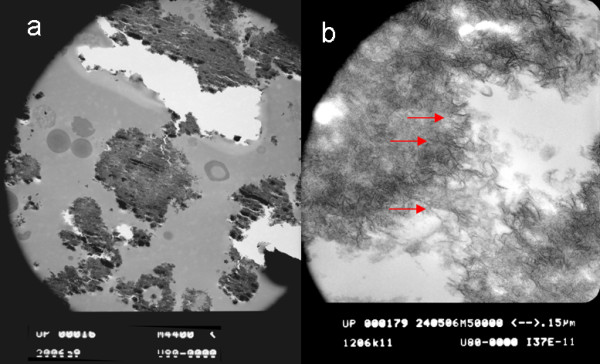
**ESC micromass cultured for 21 days in the presence of medium containing dexamethasone, ascorbic acid, and ß-glycerolphosphate (DAG)**. Transmission electron microscopy demonstrated scorching crystallites (a) and collagen fibrils (b) in the mineralized area.

**Figure 4:**
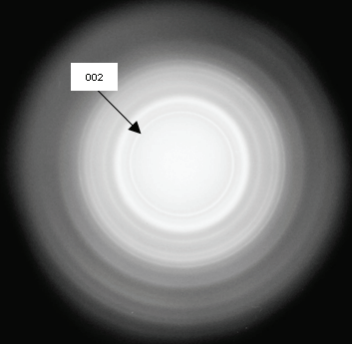
**Electron spectroscopic diffraction in the centre of DAG-treated ESC microspheres showed typical patterns for hydroxyl apatite formation (day 21)**.

After three days all cell cultures formed spheroid, three-dimensional cell units in high density (5 × 10^6 ^cells/ml), which appeared as oval micromasses. At that time, neither in specimens from the DAGstimulated group nor in the non-stimulated group signs of mineralization were detectable. After 10 days of cultivation the first indications of mineralization were visible in the DAG-treated cells, while they were absent in non-stimulated cells. Mineralization proceeded in the centre of the stimulated specimens and became more clearly visible after 3 weeks of cultivation in the presence of osteoinductive stimuli. Numerous living cells were detected in the mineralized centre of the spheres by means of toluidine blue staining (Figure 1). Generally, the mineralization was most prominent in the centre of the sphere, as demonstrated in histological sections stained with alizarin red (Figure 1). The SEM analysis confirmed the differences regarding the distribution pattern of the formed mineral and the quantitative differences. The + DAG group showed an intense mineralization in the centre of the spheres (Figure 2). Transmission electron microscopy (TEM) confirmed the presence of scorching crystallites in the mineralized area, which appeared after 21 days of cultivation (Figure 3a). Theses crystals were typically embedded in an extracellular matrix containing numerous collagenous fibrils (Figure 3b). The spherical cell units had a peripheral zone consisting of densely packed cell layers, which surrounded the minerals. To demonstrate that the mineralized matrix in the DAG-treated group is composed of hydroxyl apatite crystals, electron spectroscopic diffraction analysis was performed (Figure 4). In accordance with Arnold et al. and Plate et al., the d-value for the diffraction ring 002 was calculated (0.344 nm) (Figure 3 and 4).

## Discussion

The de-novo formation of bone in terms of tissue engineering requires cells, matrix and growth factors. For creating larger tissue constructs for surgical use, natural or artificial biomaterials are additionally needed as scaffolds. However, there is controversy about the use of biomaterials as a scaffold because the physicochemical properties of the biomaterials influence the proliferation and gene expression of the cells [9,21,22]. Even protein coating of the scaffold has impact on the attachment of the cells [23-25]. It is generally accepted that no existing artificial or natural scaffold can meet all the requirements for ruling out undesired effects. The micromass culture technique may be an alternative for substituting artificial scaffolds. In contrast to monolayers, cell culture-based techniques in three-dimensional space appear to more closely resemble in-vivo conditions [11]. It is well known that many functions of the cells, e.g. differentiation and proliferation, rely on intact cell-cell interactions and a tight attachment to extracellular matrix components. In micromasses, the cells can interact with each other and maintain these interactions [26]. Former studies have shown that in micromass culture techniques a cartilaginous differentiation of ESCs is feasible [27,28]. In the presented study we show that stimulated ESCs cultured in micromass technique form minealized microspheres during cultivation.

Aggregation of cells is the pivotal stage in the development of skeletal tissues and the primary resource from which the skeleton is built and through which the skeleton is modified ontogenetically [29]. Mineralized bony units formed ex vivo seem to be an ideal biomaterial because they combine the structural features of bone. Currently, the best treatment option for bone defects utilises the enhanced regeneration potential of embryonic stem cells [30]. In this respect, fusion of multiple bony units may allow the reconstruction of larger skeletal elements. Through the ability of embryonic stem cells to differentiate along the whole osteogenic pathway, embryonic stem cell transplantation may play a future role in the treatment of generalized bone diseases. Furthermore, we show that osteoinductive stimuli including DAG support the mineralization of the extracellular matrix and that stimulated micromasses produce more mineralized extracellular matrix than micromasses cultured in the absence of these stimuli. To verify that the matrix consists of hydroxyl apatite, we performed transmission electron microscopy and revealed a time-dependent occurrence of scorching crystallites in the interior of the microspheres. Using electron spectroscopic diffraction we confirmed that the crystallites consisted of hydroxyl apatite. Furthermore, we detected collagen fibrils that were morphologically very similar to collagenous fibrils within bone tissue. Collagen I fibrils are known to be a major extracellular matrix component of bone tissue [5,31]. Plate and co-workers described the formation of hydroxyl apatite in bone and dentin as a multistage process resulting in the deposition of a mineralized matrix. These calcium-phosphate crystals coordinate longitudinally and accumulate as scorching crystallites [20]. In samples from stimulated ESCs we detected crystal-like structures in the interior of the microspheres. These ESC microspheres resemble aggregates consisting of preosteoblasts.

Our finding of a mineralization in microspheres of DAG-treated ESCs seems to share similarity to the formation of bone and dentin in vivo. Thus, it appears that osteologous differentiation of ESC micromasses may be a feasible approach to advance the bony reconstruction of large defects. However, the size of the microspheres is limited possibly due to restricted diffusion of nutrients and we are currently unable to format larger tissue constructs without support by artificial matrices. The use of bioreactors may be an adequate technique to gain larger tissue constructs without the need for a scaffold by simply transferring osteologously differentiated ECS micromasses [32].

Nevertheless, the micromass culture technique may be an appropriate model to analyse the formation of the skeleton during embryonic or fetal organogenesis. Aggregation of cells to a critical size is a fundamental step in initiating organogenesis of vertebrates [33]. Hall and Miyake assume that the condensation of cells is a precondition for skeleton formation that promotes the differentiation of cells to osteoblasts and chondroblasts [29,34]. Furthermore, the three-dimensional micromass culture technique may be a useful method for identifying substances that enhance mineralization.

The use of embryonic stem cells will probably play a major role in tissue engineering in the future because of the remakable potential and differentiation capacity of ESCs. Prior to clinical application, many challenges need to be faced in future studies, particularly with respect to immune tolerance and the formation of malignant tumors in the host organism. However, the studies by Burt and coworkers are promising with regard to immuntolerance. They grafted ESCs into MHC-mismatched mice and found no clinical or histological evidence for a graft-versus-host or host-versus-graft reaction [35].

Furthermore, Zavazava has demonstrated that ESCs have the potential to induce immune tolerance [36] and revealed evidence for a suppression of the MHC gene expression [37]. Trounson and colleagues showed that transplanted undifferentiated ESCs may induce teratoma and teratocarcinoma [38]. Even if many other authors could not find any indication of malignant transformation in their studies [39], the eventuality of cancer induction is still an argument for the restricted use of these cells. Lastly, there are legal and ethical restrictions for the use of human ESCs.

Despite the above mentioned doubts about the use of ESCs, they may open up new therapeutic options for future application and may turn out to be interesting models for the study of fetal organogenesis. Furthermore, the results may be transferred to other pluripotent stem cells, such as umbilical somatic stem cells, which have not so many restrictions.

## Competing interests

The authors declare that they have no competing interests.

## Authors' contributions

All authors have read and approved the final manuscript.
